# Feeding Stimulates Sphingosine-1-Phosphate Mobilization in Mouse Hypothalamus

**DOI:** 10.3390/ijms20164008

**Published:** 2019-08-17

**Authors:** Valentina Vozella, Natalia Realini, Alessandra Misto, Daniele Piomelli

**Affiliations:** 1 Department of Anatomy and Neurobiology, University of California, Irvine, CA 92697, USA; 2 Fondazione Istituto Italiano di Tecnologia, Via Morego 30, 16163 Genova, Italy; 3 Department of Biological Chemistry, University of California, Irvine, CA 92697, USA; 4 Center for the Study of Cannabis, University of California, Irvine, CA 92697, USA

**Keywords:** Sphingolipids, hypothalamus, feeding, sphingosine-1-phosphate, sphinganine-1-phosphate

## Abstract

Previous studies have shown that the sphingolipid-derived mediator sphingosine-1-phosphate (S1P) reduces food intake by activating G protein-coupled S1P receptor-1 (S1PR1) in the hypothalamus. Here, we examined whether feeding regulates hypothalamic mobilization of S1P and other sphingolipid-derived messengers. We prepared lipid extracts from the hypothalamus of C57Bl6/J male mice subjected to one of four conditions: free feeding, 12 h fasting, and 1 h or 6 h refeeding. Liquid chromatography/tandem mass spectrometry was used to quantify various sphingolipid species, including sphinganine (SA), sphingosine (SO), and their bioactive derivatives SA-1-phosphate (SA1P) and S1P. In parallel experiments, transcription of S1PR1 (encoded in mice by the *S1pr1* gene) and of key genes of sphingolipid metabolism (*Sptlc2*, *Lass1*, *Sphk1*, *Sphk2*) was measured by RT-PCR. Feeding increased levels of S1P (in pmol-mg^−1^ of wet tissue) and SA1P. This response was accompanied by parallel changes in SA and dihydroceramide (d18:0/18:0), and was partially (SA1P) or completely (S1P) reversed by fasting. No such effects were observed with other sphingolipid species targeted by our analysis. Feeding also increased transcription of *Sptlc2*, *Lass1*, *Sphk2*, and *S1pr1*. Feeding stimulates mobilization of endogenous S1PR1 agonists S1P and SA1P in mouse hypothalamus, via a mechanism that involves transcriptional up-regulation of de novo sphingolipid biosynthesis. The results support a role for sphingolipid-mediated signaling in the central control of energy balance.

## 1. Introduction

**Figure 1:**
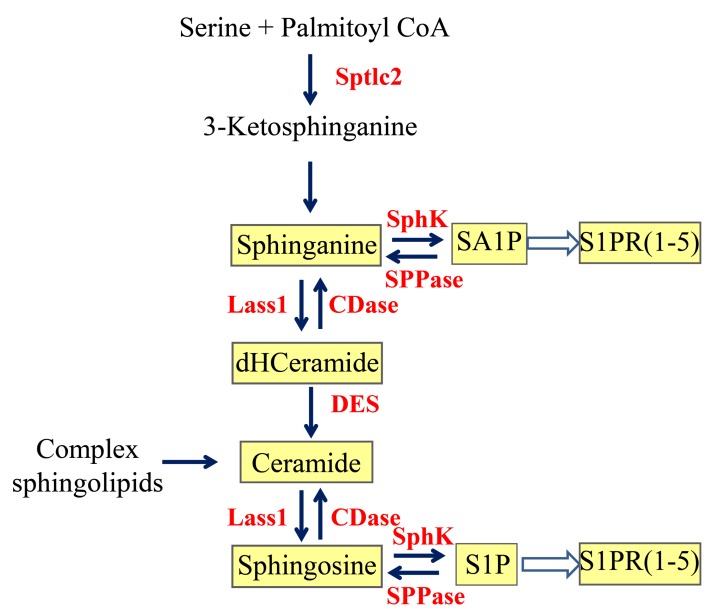
Formation of bioactive sphingosine-1-phosphate (S1P) and sphinganine-1-phosphate (SA1P) in mammalian cells. Sptcl2: serine palmitoyltransferase long chain base subunit 2; Lass1: longevity assurance gene 1; CDase: ceramidase; DES: desaturase; SphK: sphingosine/sphinganine kinase; SPPase: sphingosine/sphinganine-1-phosphate phosphatase; S1PR: sphingosine/sphinganine-1-phosphate receptor; dHCeramide: dihydroceramide.

The hypothalamus controls feeding behavior and energy metabolism by integrating a complex array of neural and humoral signals [1,2,3,4]. Peptide neurotransmitters are known to play crucial roles in this process. For example, neurons housed in the hypothalamic arcuate nucleus secrete either orexigenic or anorexic neuropeptides [5], such as agouti-related protein (AgRP) [6] and α-melanocyte-stimulating hormone (α-MSH) [7], which interact functionally to regulate feeding and maintain energy homeostasis. While the functions served by peptide transmitters in the central control of feeding behavior are well established, much less is known about the roles that lipid-derived mediators may play in this process. A recent study suggested that sphingolipids—a quantitatively major but still poorly understood lipid class in the mammalian brain [8]—contribute to the hypothalamic regulation of energy homeostasis [9]. In this study, Silva and collaborators reported that intracerebroventricular infusions of the bioactive sphingoid base sphingosine-1-phosphate (S1P) decrease food intake, whereas genetic disruption of S1PR1, the G protein-coupled receptor engaged by S1P, exerts an opposite effect [9]. These authors also demonstrated that S1PR1 is expressed in regions of the hypothalamus, such as the arcuate and ventromedial/dorsomedial nuclei, which are involved in the control of feeding [9]. These data provide important pharmacological evidence for a role of sphingolipid-derived signals in the regulation of energy balance, but do not establish whether such signals are produced in the hypothalamus under physiological conditions. To address this question, in the present study we evaluated the impact of positive or negative energy balance (i.e., free feeding, fasting and refeeding) on the mobilization (formation and/or degradation) of S1P and other sphingolipids in the mouse hypothalamus. The primary pathways of S1P metabolism are illustrated in Figure 1. The results suggest that food deprivation suppresses [9]—while feeding restores—the mobilization of S1P and its bioactive analog sphinganine-1-phosphate (SA1P) through a mechanism that requires the transcriptional down-regulation of sphingolipid biosynthesis.

## 2. Results

### 2.1. Feeding Increases SA1P and S1P Levels in Mouse Hypothalamus

To determine whether feeding status influences the mobilization of sphingolipid-derived messengers in the hypothalamus, we used a liquid chromatography/mass spectrometry (LC/MS-MS) protocol that allows the simultaneous quantification of up to 25 sphingolipid species, including the sphingoid bases sphinganine (SA) and sphingosine (SO) and their bioactive phosphorylated derivatives, sphinganine-1-phoshate (SA1P) and S1P (Figure 1).

**Figure 2:**
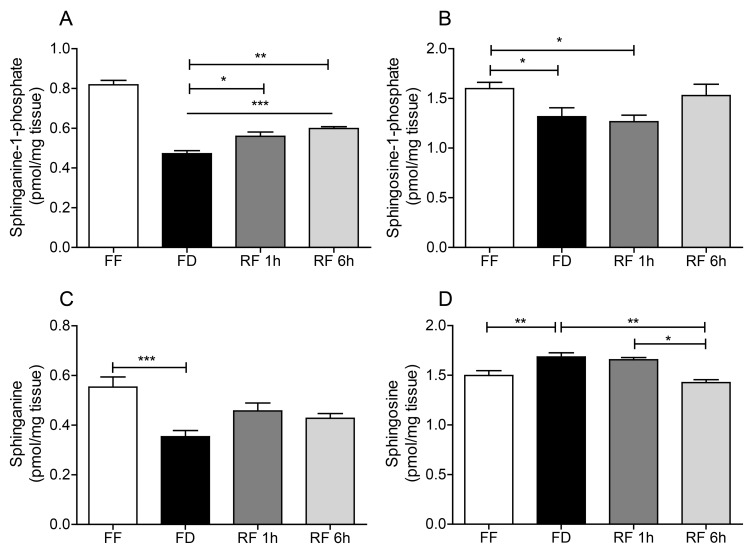
Effects of feeding status on (**A**) sphinganine-1-phosphate, (**B**) sphingosine-1-phosphate, (**C**) sphinganine and (**D**) sphingosine in mouse hypothalamus. Free feeding (FF), 12 h food deprivation (FD), 1 h refeeding after FD (RF 1h), and 6 h refeeding after FD (RF 6h). Results are expressed as mean ± SEM (*n* = 5–10/feeding condition). * *p* < 0.05, ** *p* < 0.01, *** *p* < 0.001; one-way ANOVA followed by Tukey’s multiple comparison test.

We analyzed lipid extracts of hypothalamic tissue from male C57Bl/6J mice subjected to four conditions: free feeding, 12 h food deprivation, and 12 h food deprivation followed by 1 or 6 h refeeding. The results show that fasting caused a marked decrease in the hypothalamic levels of SA1P, S1P and SA, which was partially (SA1P) or completely (S1P, SA) reversed after 6 h refeeding (Figure 2A–C). Conversely, hypothalamic SO content increased by fasting and returned to baseline levels upon refeeding (Figure 2D). The results suggest that feeding stimulates the mobilization of bioactive SA1P and S1P in mouse hypothalamus.

### 2.2. Effects of Feeding On Hypothalamic Sphingolipid Metabolism

**Figure 3:**
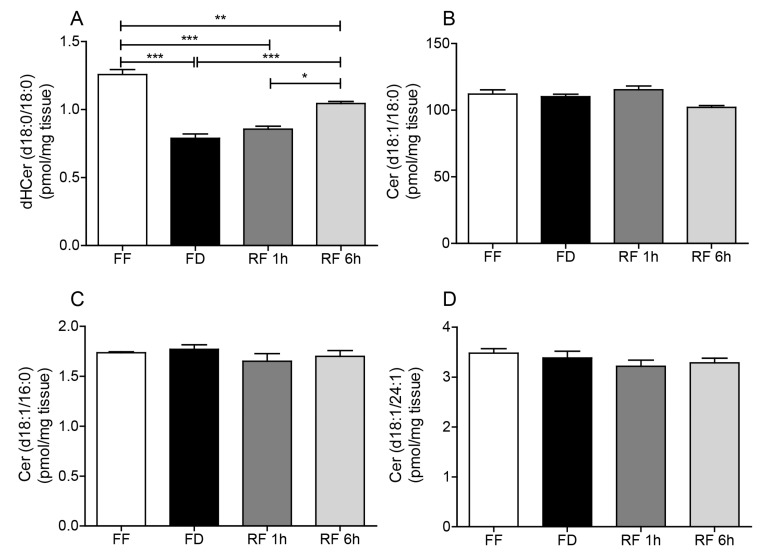
Effects of feeding status on (**A**) dihydroceramide (d18:0/18:0), (**B**) ceramide (d18:1/18:0), (**C**) ceramide (d18:1/16:0) and (**D**) ceramide (d18:1/24:1) in mouse hypothalamus. Free feeding (FF), 12 h food deprivation (FD), 1 h refeeding after FD (RF 1h) and 6 h refeeding after FD (RF 6h). Results are expressed as mean ± SEM (*n* = 5–10/feeding condition). * *p* < 0.05, ** *p* < 0.01, *** *p* < 0.001; one-way ANOVA followed by Tukey’s multiple comparison test.

We next asked whether feeding might also affect the formation of other sphingolipid species, such as dihydroceramide and ceramide, which are metabolically related to SA1P and S1P (Figure 1). LC/MS-MS analyses of hypothalamic extracts revealed that levels of dihydroceramide (d18:0/18:0), a metabolic precursor for both SA1P and S1P, were markedly lower in the hypothalamus of food-deprived mice compared to free-feeding or refed animals (Figure 3A). By contrast, no such response was observed with six additional ceramide species targeted by our analysis, including ceramide (d18:1/18:0), ceramide (d18:1/16:0), ceramide (d18:1/24:1), dihydroceramide (d18:0/16:0), dihydroceramide (d18:0/24:0), and dihydroceramide (d18:0/24:1) (Figure 3B–D, Table 1). The observed changes in sphingolipid levels raise the possibility that feeding stimulates S1P and SA1P mobilization by up-regulation of sphingolipid biosynthesis.

### 2.3. Effects of Feeding Status on Expression of Sphingolipid-Metabolizing Enzymes

**Figure 4:**
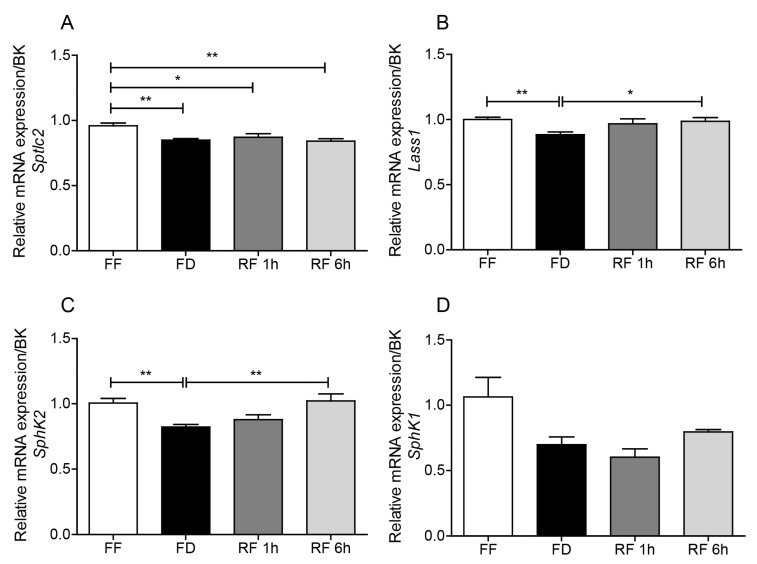
Effects of feeding status on transcription of (**A**) serine palmitoyltransferase long chain base subunit 2 (*Sptlc*2), (**B**) ceramide synthase 1 (*Lass1*), (**C**) sphingosine/sphinganine kinase 2 (*SphK2*), (**D**) sphingosine/sphinganine kinase 1 (*SphK1*). Free feeding (FF), 12 h food deprivation (FD), 1 h refeeding after FD (RF 1h) and 6 h refeeding after FD (RF 6h). Results are expressed as mean ± SEM (*n* = 5–8/feeding condition). * *p* < 0.05, ** *p* < 0.01, *** *p* < 0.001; one-way ANOVA followed by Tukey’s multiple comparison test. BK: BestKeeper.

As a direct test of this hypothesis, we evaluated the impact of feeding status on the transcription of four genes involved in sphingolipid production: *Sptlc2*, *Lass1*, *SphK1, and SphK2* (Figure 1). *Sptlc2* (serine palmitoyltransferase long chain base subunit 2) encodes for the long-chain base subunit of the enzyme serine palmitoyltransferase, which mediates the first committed step in sphingolipid biosynthesis [10]. *Lass1* (longevity assurance gene 1) encodes for ceramide synthase 1, which catalyzes the biosynthesis of 18-carbon ceramides in brain neurons [11]. *SphK1* (sphinganine/sphingosine kinase 1) and *SphK2* (sphinganine/sphingosine kinase 2) encode for intracellular lipid kinases that convert SO and SA into S1P and SA1P. SphK1 shows greater selectivity for SO and SphK2 for SA [12], and both isoforms are expressed in the mouse brain [13]. Quantitative RT-PCR analyses show that fasting is accompanied by significant decreases in the transcription of *Sptlc2*, *Lass1,* and *SphK2*, which are partially (*Sptlc2*) or completely (*Lass1*, *SphK2*) reversed by refeeding (Figure 4A–C). A similar, albeit non-significant, trend was also observed with *SphK1* (Figure 4D). Together, our findings suggest that food intake can influence SA1P and S1P mobilization, at least in part, via transcriptional regulation of de novo sphingolipid biosynthesis.

### 2.4. Feeding Regulates S1pr1 Transcription in Hypothalamus

**Figure 5:**
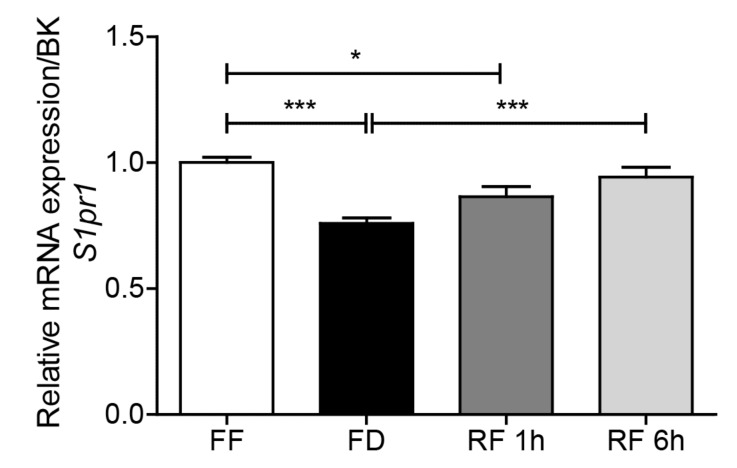
Effects of feeding status on transcription of sphingosine-1-phosphate receptor 1 (*S1pr1*). Free feeding (FF), 12 h food deprivation (FD), 1 h refeeding after FD (RF 1h) and 6 h refeeding after FD (RF 6h). Results are expressed as mean ± SEM (*n* = 5–8/feeding condition). * *p* < 0.05, ** *p* < 0.01, *** *p* < 0.001; one-way ANOVA followed by Tukey’s multiple comparison test. BK: BestKeeper.

The biological effects of S1P and SA1P are mediated by activation of the G protein-coupled receptor S1PR1-5 [14,15]. Previous studies have shown that fasting down-regulates S1PR1 protein levels in rat hypothalamus [9]. Quantitative RT-PCR analyses of hypothalamic tissue from mice maintained under free feeding, fasting or refeeding conditions confirmed this finding: *S1pr1* transcription levels were significantly reduced after 12 h of fasting, an effect that was rapidly and completely reversed by refeeding (Figure 5).

## 3. Discussion

Pharmacological experiments in rats have shown that intracerebroventricular infusions of the sphingolipid-derived mediator S1P produce a behaviorally selective inhibition of food intake by activating G protein-coupled S1PR1 receptors in the hypothalamus [9]. These findings suggest that S1P may act as a central satiety factor, and raise the question of whether the mobilization of this lipid messenger might be regulated by the feeding status. In the present report, we show that feeding causes a marked transcriptional up-regulation of sphingolipid biosynthesis in the mouse hypothalamus, which is accompanied by enhanced local mobilization of S1P and its isofunctional analog SA1P. The results support a role for sphingolipid-mediated signaling in the hypothalamic control of food intake.

Our LC/MS-MS analyses show that feeding increases the hypothalamic content of four key intermediates in the de novo pathway of sphingolipid metabolism: SA, SA1P, dihydroceramide (d18:0/18:0) and S1P. As illustrated in Figure 1, SA is the immediate precursor for both SA1P and dihydroceramide, which is then converted via ceramide into SO and S1P. In the brain, these reactions are catalyzed by four enzymes—encoded for by the genes *Sptlc2*, *Lass1*, *SphK1* and *SphK2*—whose transcription levels are also increased in the fed state. These convergent results support the hypothesis that feeding enhances sphingolipid metabolism and, by doing so, accelerates the production of bioactive sphingolipid-derived signals in the hypothalamus. Of note, we found that ceramide (d18:1/18:0) and SO are distinct from other sphingolipids in that their levels are either unaffected (ceramide) or decreased (SO) by feeding. One possible explanation for this difference may be the concurrent recruitment of the salvage pathway, an alternate route of sphingolipid metabolism that produces ceramide and SO via cleavage of sphingomyelin or hexosylceramide [16,17,18].

The hypothalamus is a crucial hub for ‘lipid sensing’, the process that monitors circulating fatty acid levels to control food intake, insulin secretion, hepatic glucose production, and adipose storage [19,20]. In obesity and other metabolic disorders, this process may be disrupted by the toxic accumulation of various bioactive lipids, including ceramides, in the hypothalamus [21,22,23]. It has been shown that obesity and diabetes are associated with increased hypothalamic ceramide levels [24,25] and that administration of exogenous ceramide causes lipotoxicity, decreased thermogenesis and feeding-independent weight gain [23]. The present results raise the intriguing possibility that pathological alterations in hypothalamic lipid sensing, such as those documented in obese animals, may be rooted in a physiological role for sphingolipid-derived signals in the regulation of food intake. In this context, it is tempting to speculate that humoral factors, such as circulating fatty acids, might be responsible for the post-ingestive stimulation of sphingolipid metabolism and sphingoid base mobilization reported in the present study. This hypothesis will be addressed in future experiments.

In conclusion, the present study addressed the question of whether sphingolipid-derived signals are mobilized in the hypothalamus under conditions of positive or negative energy balance. The results suggest that feeding stimulates, whereas fasting suppresses the mobilization of S1P and its isofunctional analog SA1P, through a mechanism that requires the transcriptional down-regulation of sphingolipid biosynthesis. Together, the findings point to a functional role for S1P and SA1P in the central control of energy balance.

## 4. Materials and Methods

### 4.1. Animals

Male C57Bl/6J mice (8-week-old) were purchased from Charles River Laboratories (Calco, Lecco, Italy). Upon arrival, the animals were acclimatized to the vivarium and kept in a temperature (22 °C) and humidity-controlled environment under a 12 h light/12 h dark cycle (lights on at 7:00 A.M.). All procedures complied with the ARRIVE (Animal Research: Reporting of In Vivo Experiments) guidelines, the commonly accepted “3Rs” guidelines, and were performed in accordance with the Ethical Guidelines of the European Community Council (086, 24 August 2015) (Directive 2010/63/EU of 22 September 2010) and accepted by the Italian Ministry of Health.

### 4.2. Diet

Mice were fed a standard diet (2.66 kcal/g, 4RF21 GLP, Mucedola s.r.l., Settimo Milanese, Italy).

### 4.3. Chemicals

Authentic sphingolipid standards were purchased from Avanti Polar Lipids (Alabaster, Alabama, USA). LC/MS-MS-grade solvents were from Sigma-Aldrich (Milan, Italy).

### 4.4. Experimental Design: Effects of Food Deprivation and Refeeding

Four days before the experiments, mice were transferred to single-housing in bottom-wired cages and were randomly assigned to the following groups: free feeding (FF), 12 h food deprivation (FD), 1 h refeeding after food deprivation (RF 1h), and 6 h refeeding after food deprivation (RF 6h). Water was provided *ad libitum*. On day 5, the mice were food-deprived for 12 h during the dark phase. The refeeding groups were food deprived for 12 h and then allowed to feed for 1 h or 6 h. At the end of the experiment, the animals were euthanized by cervical dislocation.

### 4.5. Tissue Collection

Mice were anesthetized with isoflurane and sacrificed by cervical dislocation. Brains were removed, hypothalami were dissected on ice-cold glass plates, and flash frozen in liquid N_2_. Samples were stored at −80 °C until analyses.

### 4.6. Lipid Extraction

Lipid extractions were carried out according to a modified Bligh and Dyer procedure, as previously described [26]. Briefly, frozen hypothalamic tissue (~10 mg) was homogenized in 2 mL of methanol/chloroform (2:1 *v*/*v*) containing trifluoroacetic acid (TFA, 0.1% final concentration), and spiked with a mixture of internal standards consisting of the following odd-chain lipids: 200 nM ceramide (d18:1/17:0), 500 nM sphinganine (d17:0), sphingosine (d17:1), 400 nM sphinganine-1-phosphate (d17:0), and sphingosine-1-phosphate (d17:1). After mixing for 30 s, lipids were extracted with chloroform (0.6 mL) and extracts were washed with purified water (0.6 mL). Samples were centrifuged for 15 min at 2800× *g* at 15 °C. After centrifugation, the organic phases were collected and transferred to a new set of glass vials. To improve extraction efficiency, the samples were extracted twice. The organic phases were pooled, dried under N_2_ and residues were dissolved in 0.2 mL of methanol/chloroform (9:1 *v*/*v*). A total of 0.1 mL of the total extract was saved to measure more polar analytes such as sphinganine (SA), sphingosine (SO), and their phosphates, SA1P and S1P. The remaining solvent was evaporated under N_2_. Lipids were reconstituted in chloroform (2 mL) and fractionated using small glass columns packed with Silica Gel G (60-Å 230–400 Mesh ASTM; Whatman, Clifton, New Jersey). Ceramides were eluted with 2 mL of chloroform/methanol (9:1 *v*/*v*). The solvent was evaporated under N_2_; dried material was resuspended in 0.1 mL of methanol/chloroform (9:1 *v*/*v*) and transferred to glass vials for LC/MS-MS analyses.

### 4.7. LC/MS-MS Analyses

Sphingolipids were identified and quantified as described [26]. The following multiple reaction monitoring (MRM) transitions were used for identification and quantification: SA (d18:0) (*m/z* 302.2 > 284.2), SA1P (d18:0) (*m/z* 382.2 > 284.2), SO (d18:1) (*m/z* 300.2 > 282.2), S1P (d18:1) (*m/z* 380.3 > 264.2), ceramide (d18:1/16:0) (*m/z* 520.0 > 264.2), ceramide (d18:1/18:0) (*m/z* 548.0 > 264.2), ceramide (d18:1/20:0) (*m/z* 576.0 > 264.2), ceramide (d18:1/22:0) (*m/z* 604.3 > 264.2), ceramide (d18:1/24:0) (*m/z* 632.0 > 264.2), ceramide (d18:1/24:1) (*m/z* 630.0 > 264.2), dihydroceramide (d18:0/18:0) (*m/z* 568.5 > 550.5), dihydroceramide (d18:0/16:0) (*m/z* 540.5 > 522.5), dihydroceramide (d18:0/24:0) (*m/z* 652.5 > 634.5), dihydroceramide (d18:0/24:1) (*m/z* 650.5 > 632.5).

### 4.8. mRNA Isolation, cDNA Synthesis, and Quantitative Real-time PCR

Total RNA was extracted from tissues using TRIzol (Life Technologies, Carlsbad, California, USA) and the Ambion Purelink RNA mini-kit, as directed by the supplier (Life Technologies, Carlsbad, California, USA). Samples were rendered genomic DNA-free by treatment with DNase (PureLink DNase, Life Technologies, Carlsbad, California, USA). Reverse transcription of purified mRNA (1 µg) was carried out using SuperScript VILO cDNA synthesis kit according to the protocol (Invitrogen, Carlsbad, California, USA). First-strand cDNA was amplified using the iTaq Universal SYBR Green Supermix (Biorad, Segrate, Milan, Italy) following manufacturer’s instructions. The primer sequences were: sphingosine kinase 1 (*Sphk1*) forward: GCTTCTGTGAACCACTATGCTGG, reverse: ACTGAGCACAGAATAGAGCCGC; sphingosine kinase 2 (*Sphk2*) forward: GGTGCCAATGATCTCTGAAGCTG, reverse: CTCCAGACACAGTGACAATGCC; sphingosine-1-phosphate receptor 1 (*S1pr1*) forward: CGCAGTTCTGAGAAGTCTCTGG, reverse: GGATGTCACAGGTCTTCGCCTT; serine palmitoyltransferase long chain base subunit 2 (*Sptlc2*) forward: CCAGACTGTCAGGAGCAACCAT, reverse: CTTCTTGTCCGAGGCTGACCAT; ceramide synthase 1 (*Lass1*) forward: TCTGCTGTTGCTCCTGATGGTC, reverse: CTTGGCTGTCTGAGCTTCCAGA. Quantitative PCR was performed in 96-well PCR plates. Real-time PCR reactions were run using ViiA™7 Real-Time PCR detection system (Thermo Fisher Scientific, Monza, Itay). Thermal cycling conditions were: 95 °C for 10 min, followed by 40 cycles, each cycle consisting of 15 s at 95 °C and 1 min at 60 °C. The BestKeeper software (Version1, 2003, gene-quantification, Germany) [27] was used to determine the expression stability and the geometric mean of two different housekeeping genes (glyceraldehyde 3-phosphate dehydrogenase, *Gapdh*, and hypoxanthine phosphoribosyltransferase, *Hprt*). The relative expression of genes of interest was measured by the 2^−ΔΔC*t*^ method [28], where ΔC*t* was calculated by subtracting the C*t* value of the geometric mean of the housekeeping genes from the C*t* value of the gene of interest. Data for FD, RF 1 h, and RF 6 h are reported as fold change relative to FF.

### 4.9. Statistical Analyses

Results are expressed as mean ± standard error of the mean (SEM). Comparisons of parameters between groups were made using one-way ANOVA followed by Tukey’s multiple comparison *post-hoc* test, as appropriate. GraphPad Prism software (V5.03, Inc, San Diego, California USA) was used. Differences between groups were considered statistically significant if *p* < 0.05.

## Figures and Tables

**Table 1:** Effects of feeding status on ceramide (d18:1/20:0), ceramide (d18:1/22:0), ceramide (d18:1/24:0), dihydroceramide (d18:0/16:0), dihydroceramide (d18:0/24:0), dihydroceramide (d18:0/24:1) in mouse hypothalamus.

	FF	FD	RF 1h	RF 6h
	(pmol/mg tissue)	(pmol/mg tissue)	(pmol/mg tissue)	(pmol/mg tissue)
Ceramide (d18:1/20:0)	4.20 ± 0.37	4.37 ± 0.46	4.01 ± 0.27	3.98 ± 0.36
Ceramide (d18:1/22:0)	2.65 ± 0.16	2.74 ± 0.17	2.51 ± 0.12	2.51 ± 0.14
Ceramide (d18:1/24:0)	1.24 ± 0.04	1.23 ± 0.01	1.13 ± 0.04	1.25 ± 0.06
Dihydroceramide (d18:0/16:0)	0.13 ± 0.012	0.12 ± 0.01	0.11 ± 0.01	0.12 ± 0.01
Dihydroceramide (d18:1/24:0)	0.19 ± 0.01	0.18 ± 0.01	0.17 ± 0.01	0.19 ± 0.03
Dihydroceramide (d18:0/24:1)	0.31 ± 0.02	0.27 ± 0.01	0.27 ± 0.03	0.26 ± 0.02

Free feeding (FF), 12 h food deprivation (FD), 1 h refeeding after FD (RF 1h) and 6 h refeeding after FD (RF 6h). Results are expressed as mean ± SEM (*n* = 5/feeding condition). * *p* < 0.05, ** *p* < 0.01, *** *p* < 0.001, one-way ANOVA followed by Tukey’s multiple comparison test.

## Abbreviations

S1P	sphingosine-1-phosphate
SA1P	sphinganine-1-phosphate
S1PR1	sphingosine-1-phosphate receptor 1
SO	sphingosine
SA	sphinganine
*Sptlc2*	serine palmitoyltransferase long chain base subunit 2
*Lass1*	longevity assurance gene 1
*Sphk1*	sphingosine kinase 1
*Sphk2*	sphingosine kinase 2
AgRP	agouti-related protein
α-MSH	α-melanocyte-stimulating hormone
LC/MS	liquid chromatography/mass spectrometry
FF	free feeding
FD	food deprivation
RF 1h	1 h refeeding
RF 6h	6 h refeeding
TFA	trifluoroacetic acid
MRM	multiple reaction monitoring
SEM	standard error of the mean
*Gapdh*	glyceraldehyde 3-phosphate dehydrogenase
*Hprt*	hypoxanthine phosphoribosyltransferase
